# Supplementary material for: Discovering Anticancer Effects of Phytochemicals on MicroRNA in the Context of Data Mining
Source: Nutrients. 2025 Dec 14;17(24):3913. doi: 10.3390/nu17243913 (PMC12735441; doi:10.3390/nu17243913)
Supplement: Supplementary file 1 [file nutrients-17-03913-s001.zip › nutrients-3987262-Supplementary Figures.pdf]

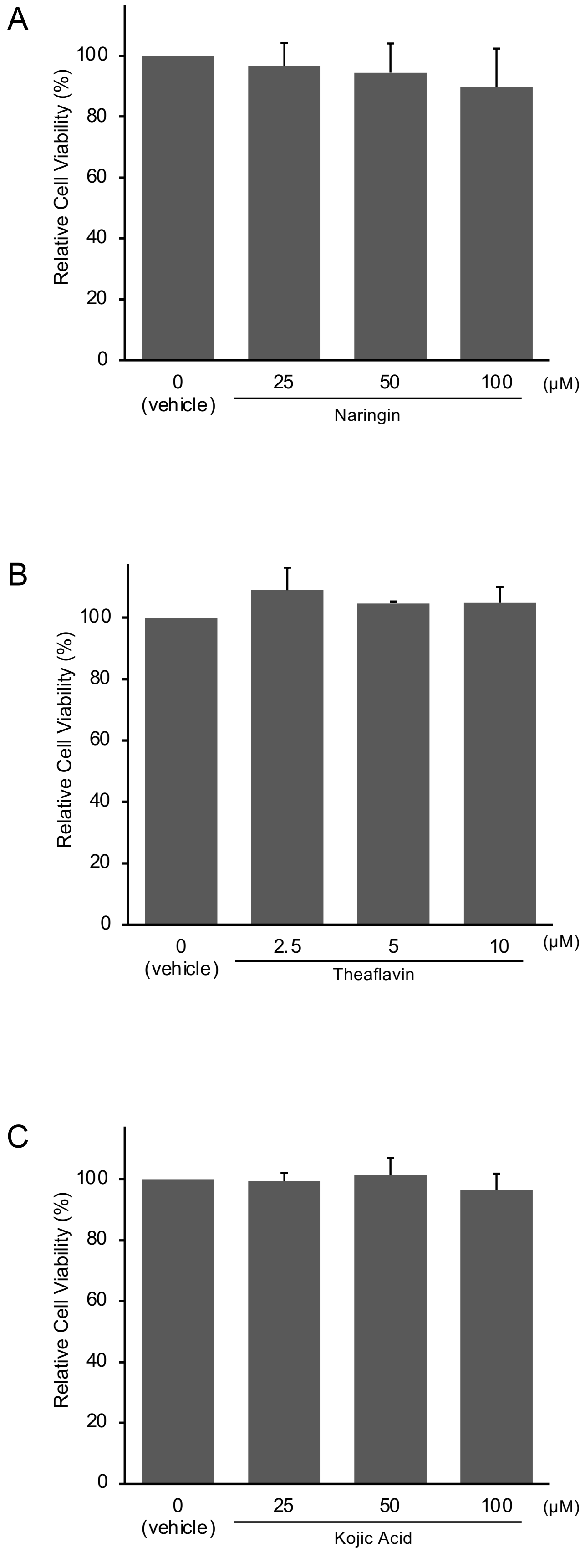

Figure S1. Effect of phytochemicals on the cell proliferation of HCT116 colon cancer cells.
A: Naringin, B: Theaflavin, and C: Kojic acid. (mean ± SD)

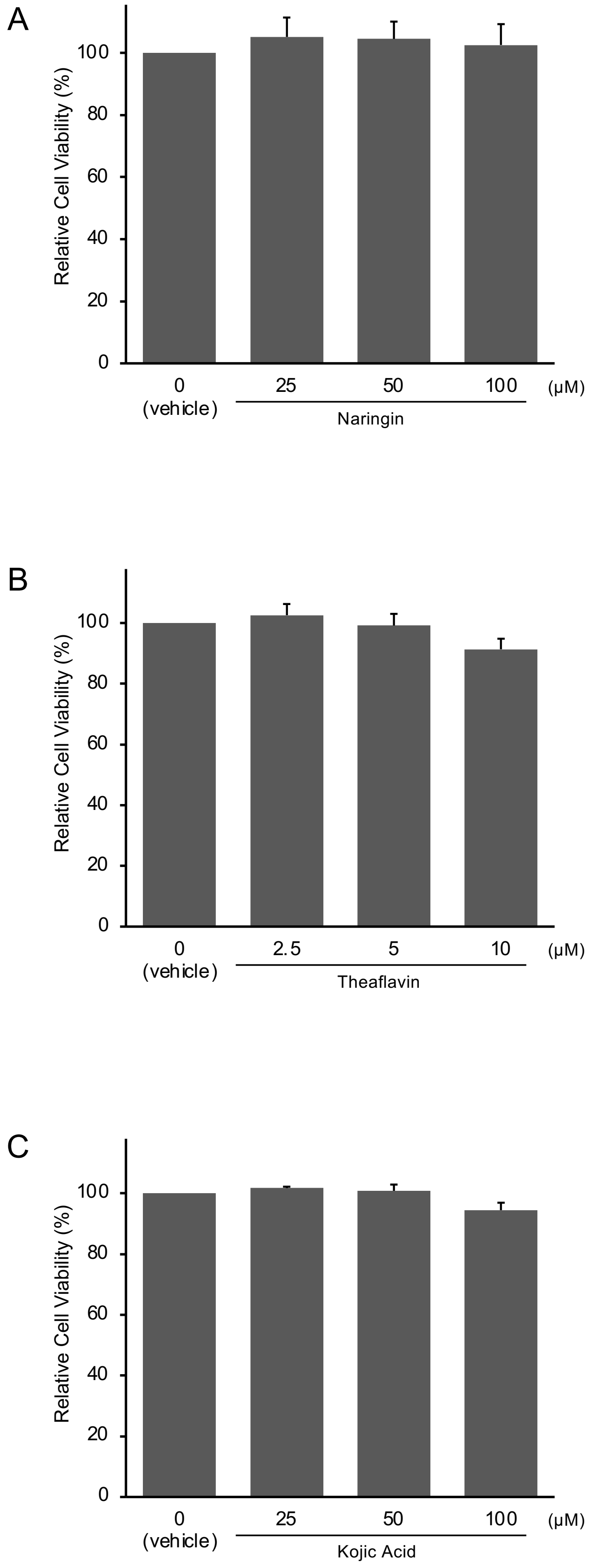

Figure S2. Effect of phytochemicals on the cell proliferation of HT29 colon cancer cells.
A: Naringin, B: Theaflavin, and C: Kojic acid. (mean ± SD)

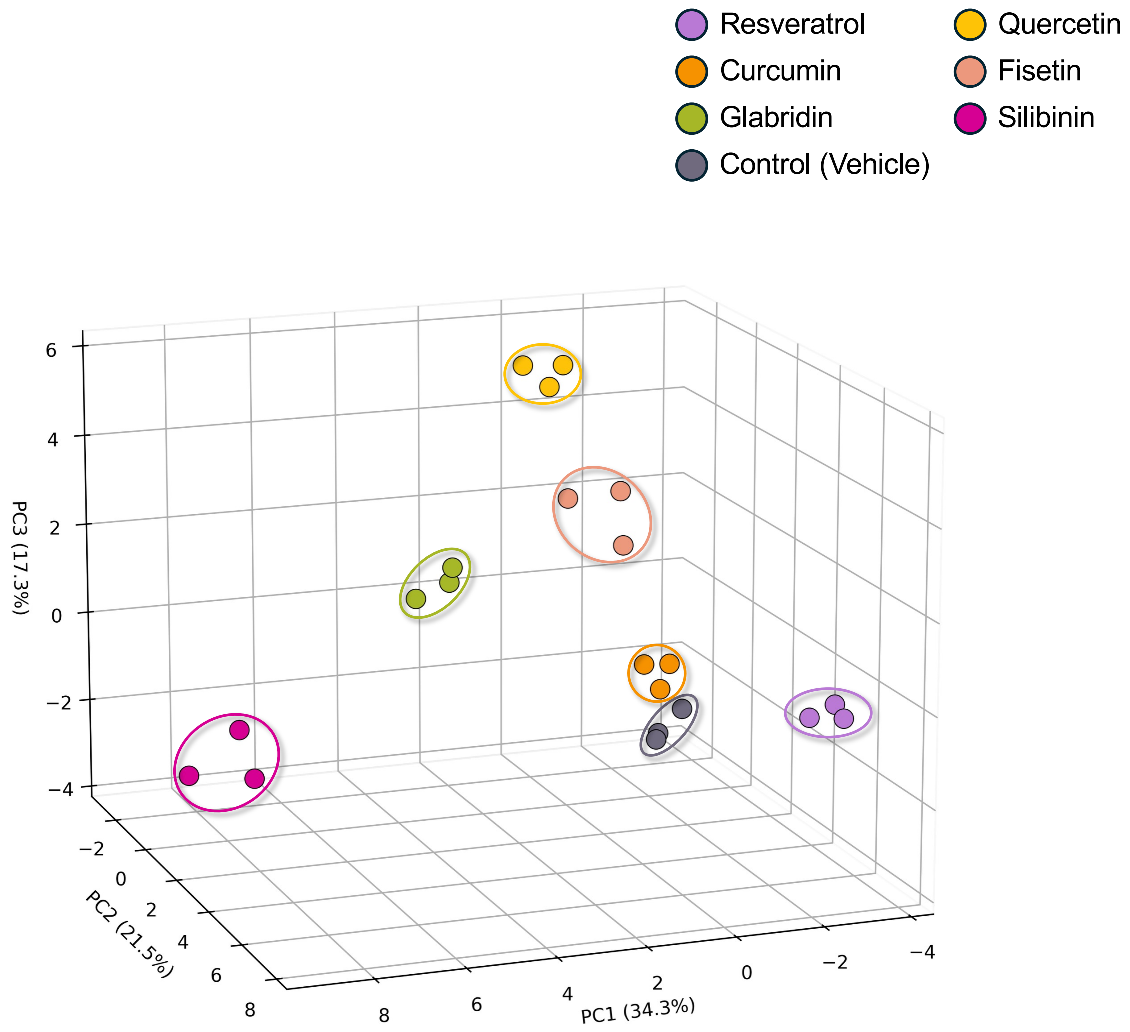

Supplemental Figure S3. Principal component analysis of miRNA expression profiles in HCT116 cells treated with phytochemicals.

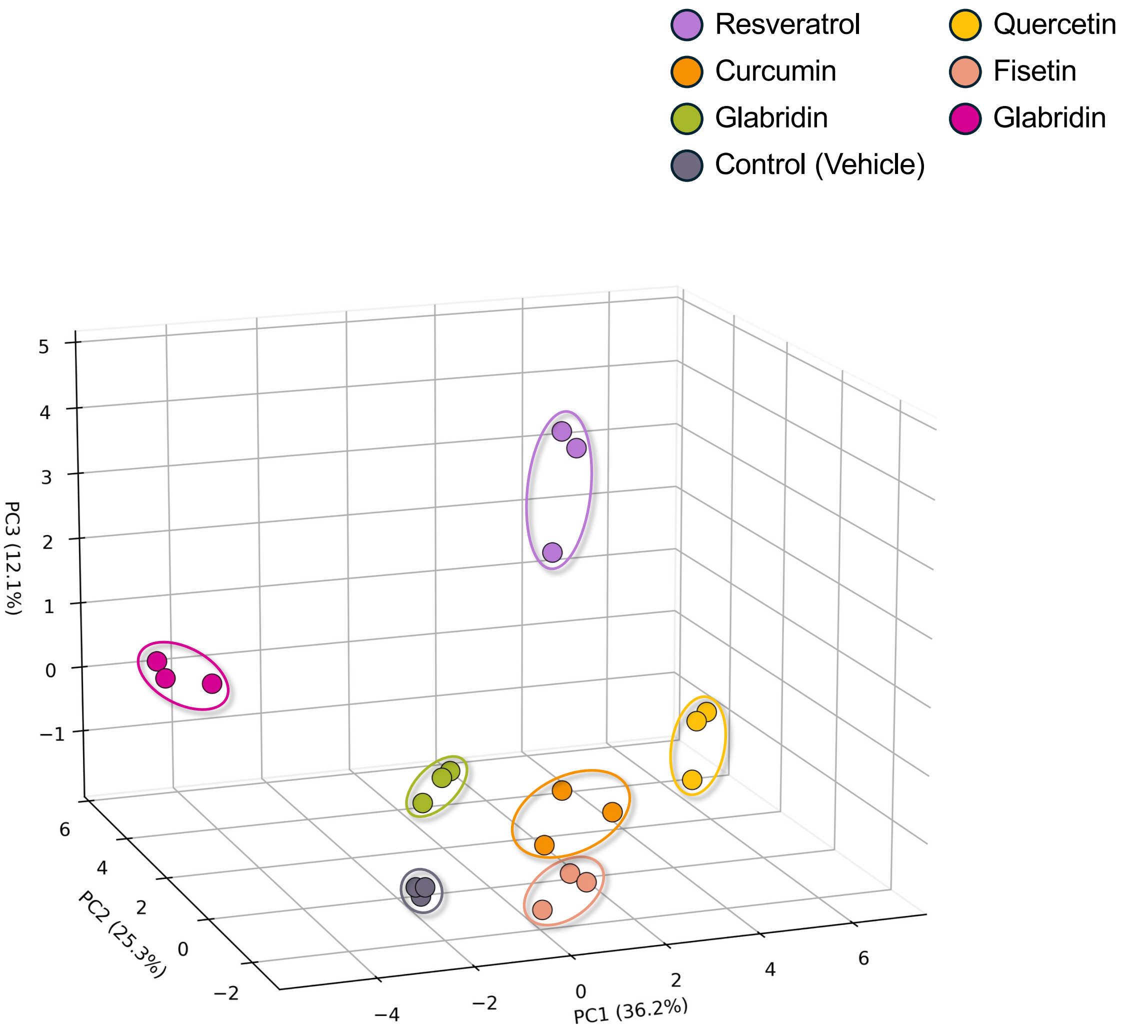

Supplemental Figure S4. Principal component analysis of miRNA expression profiles in HT29 cells treated with phytochemicals.

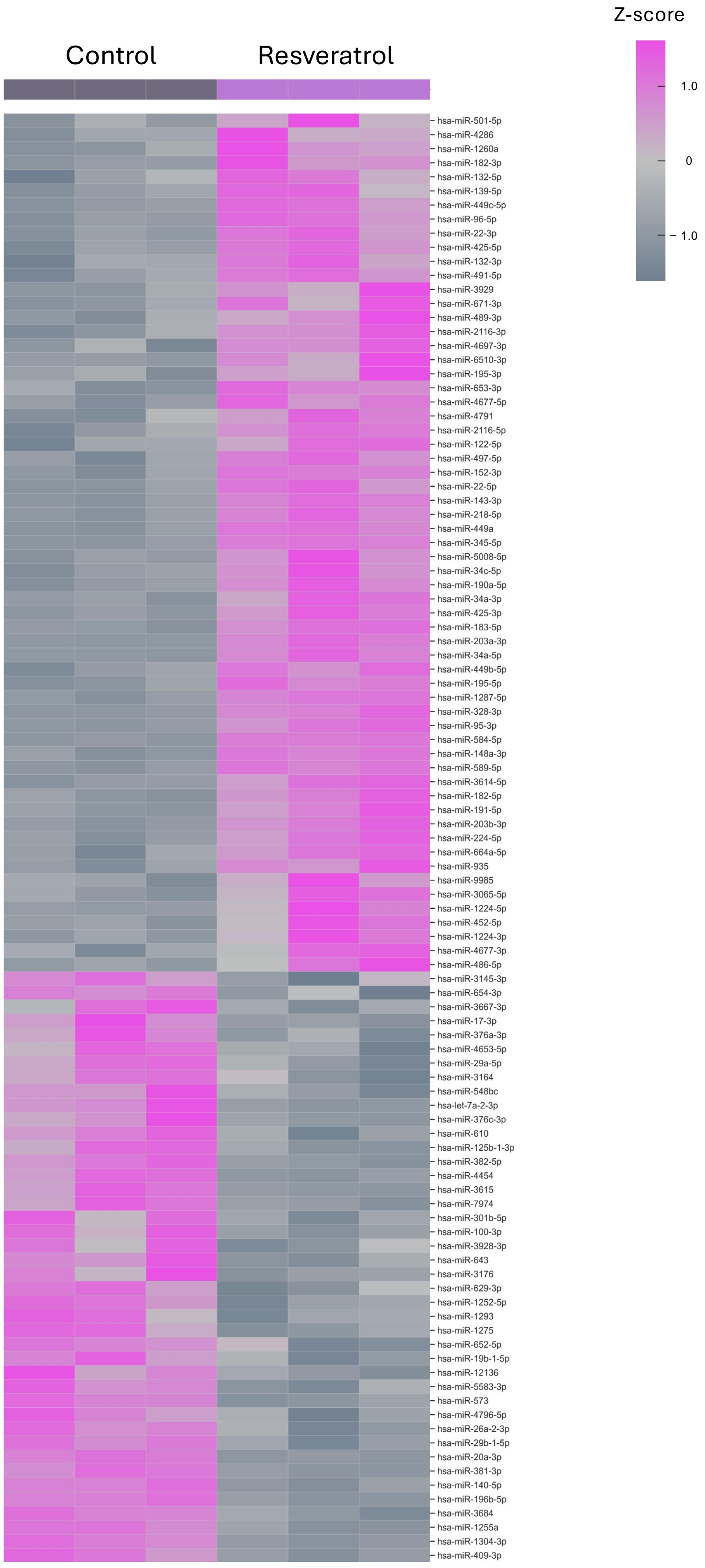

Figure S5. Heat map showing miRNAs whose expression levels differed by more than 1.5-fold in response to resveratrol treatment in HCT116 cells ($P < 0.05$).

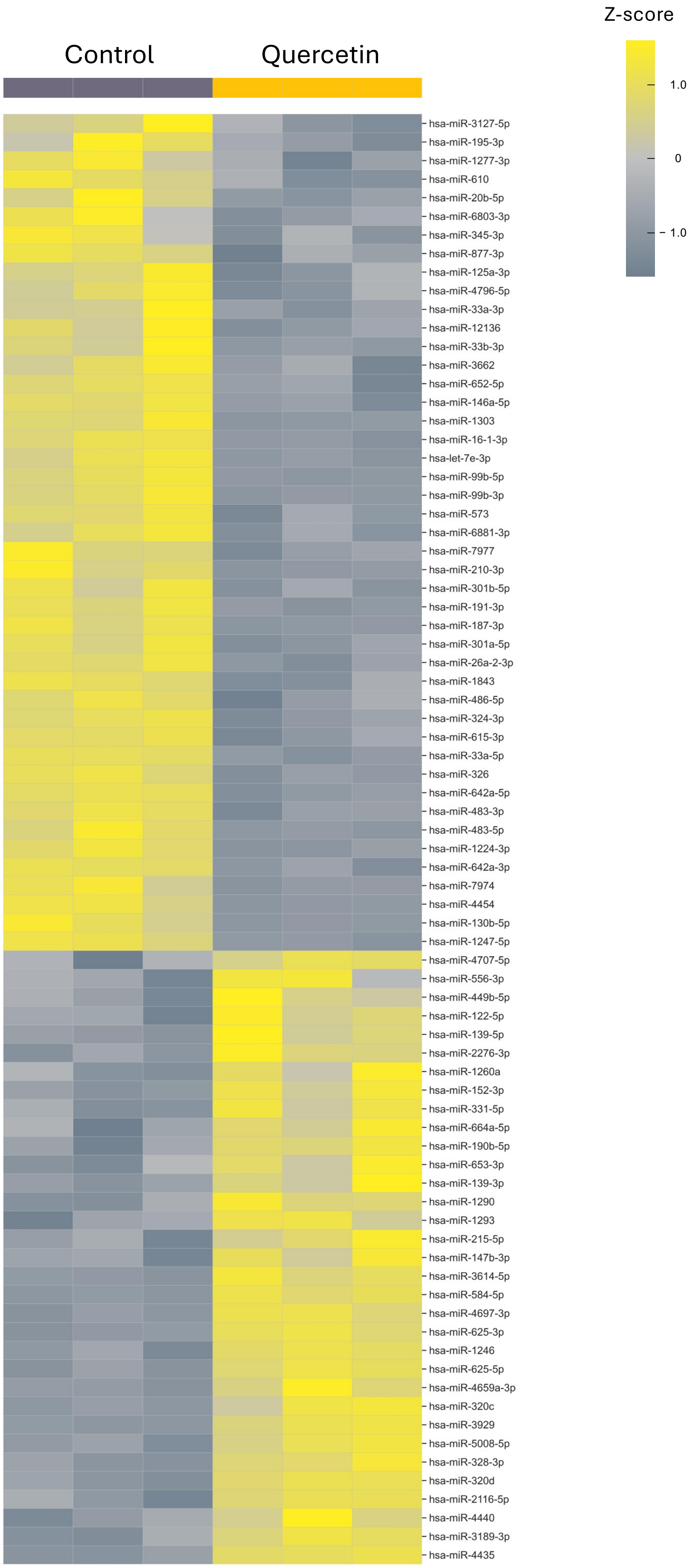

Figure S6. Heat map showing miRNAs whose expression levels differed by more than 1.5-fold in response to quercetin treatment in HCT116 cells ($P < 0.05$).

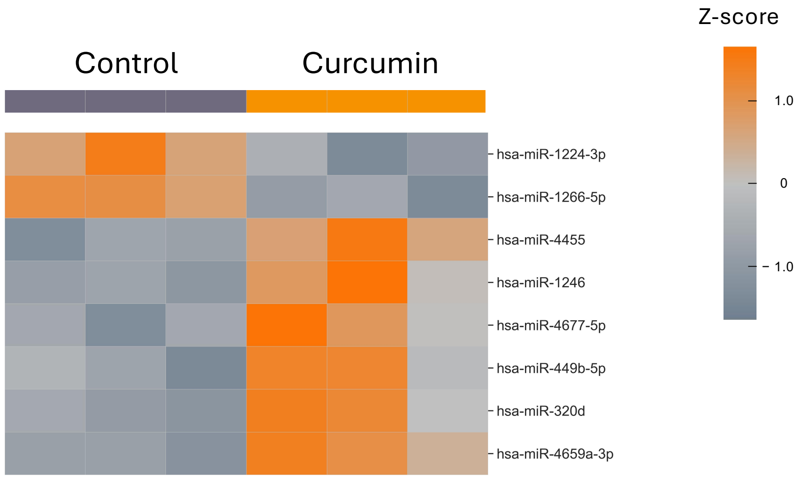

Figure S7. Heat map showing miRNAs whose expression levels differed by more than 1.5-fold in response to curcumin treatment in HCT116 cells (*P* < 0.05).

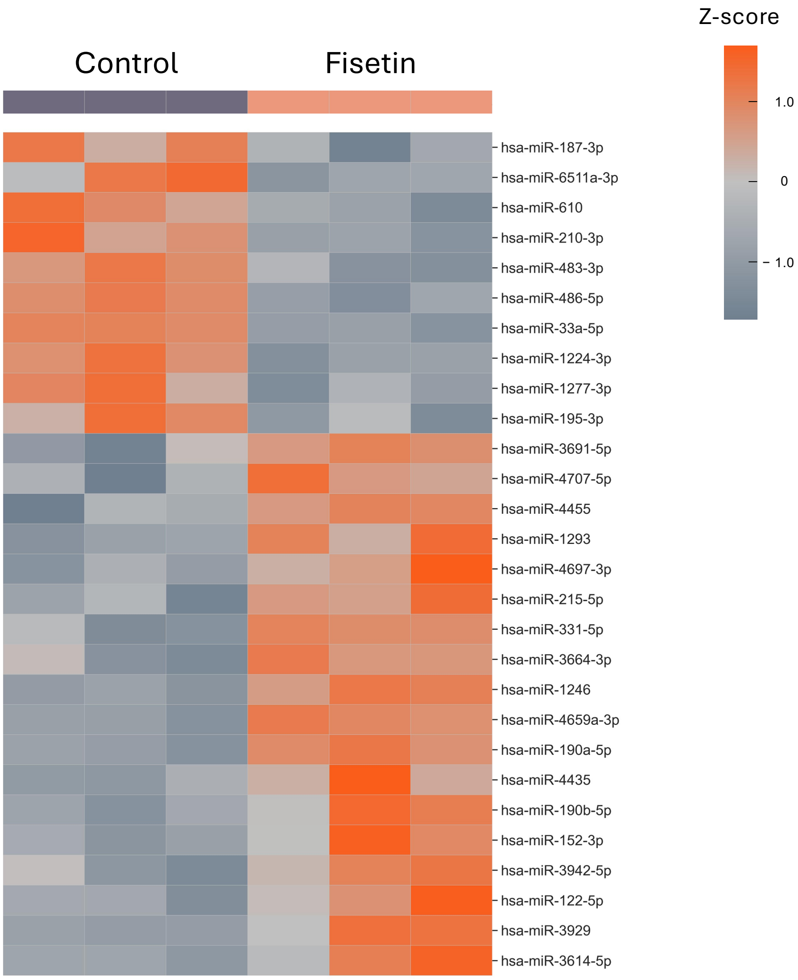

Figure S8. Heat map showing miRNAs whose expression levels differed by more than 1.5-fold in response to fisetin treatment in HCT116 cells (*P* < 0.05).

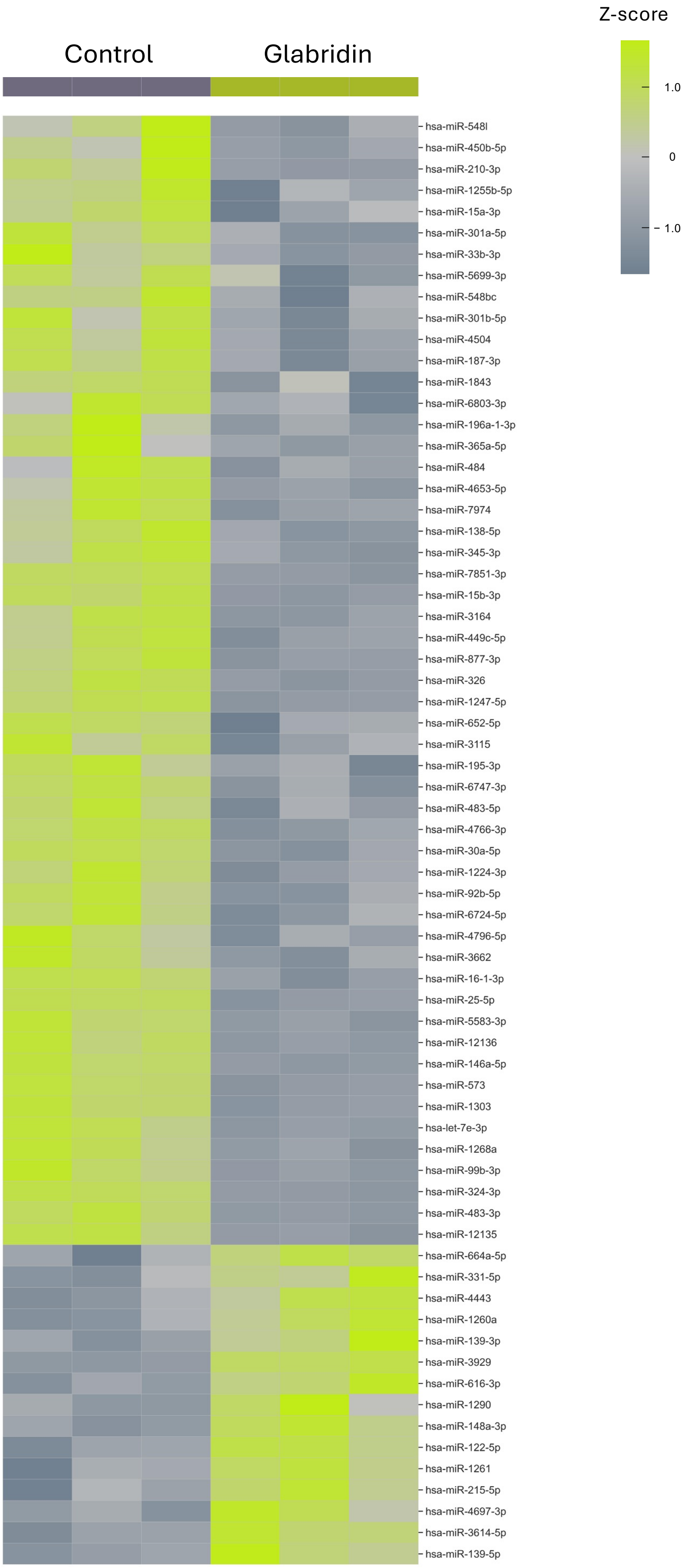

Figure S9. Heat map showing miRNAs whose expression levels differed by more than 1.5-fold in response to glabridin treatment in HCT116 cells ($P < 0.05$).

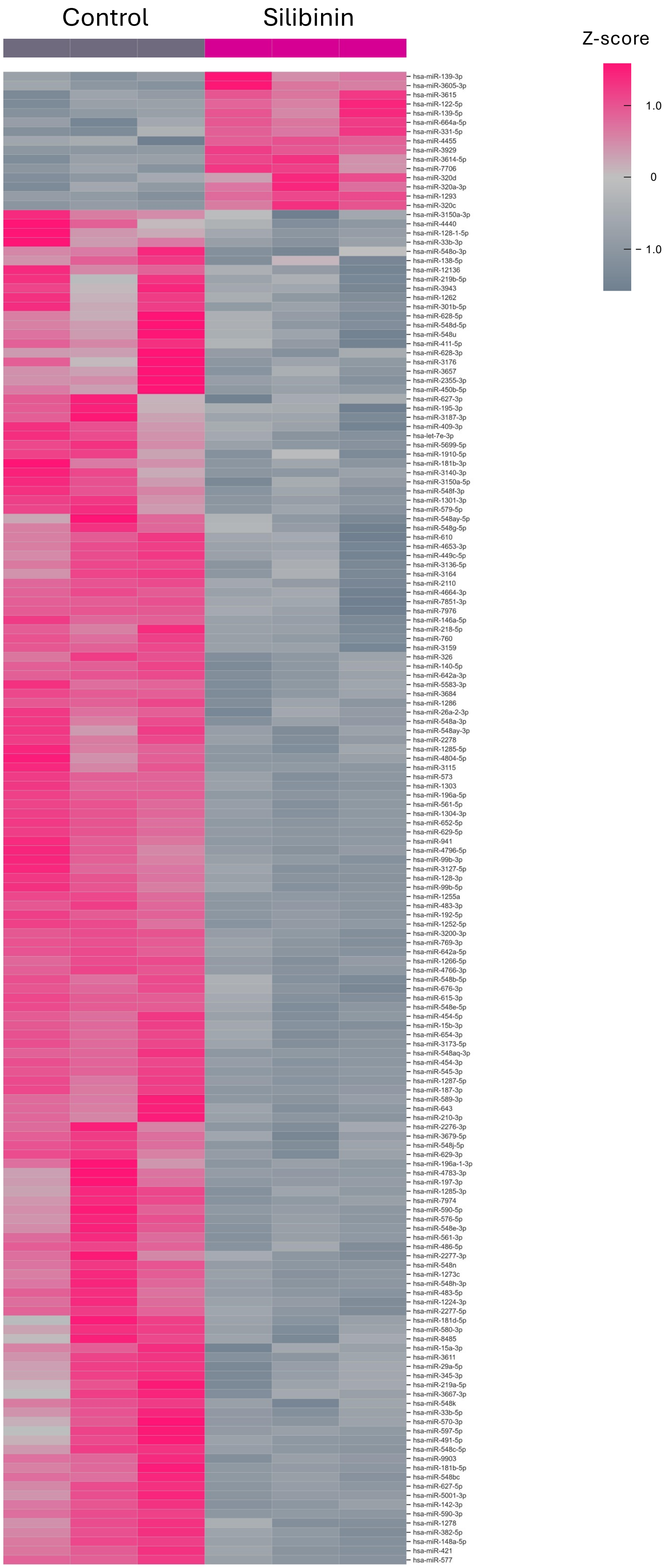

Figure S10. Heat map showing miRNAs whose expression levels differed by more than 1.5-fold in response to silibinin treatment in HCT116 cells ($P < 0.05$).

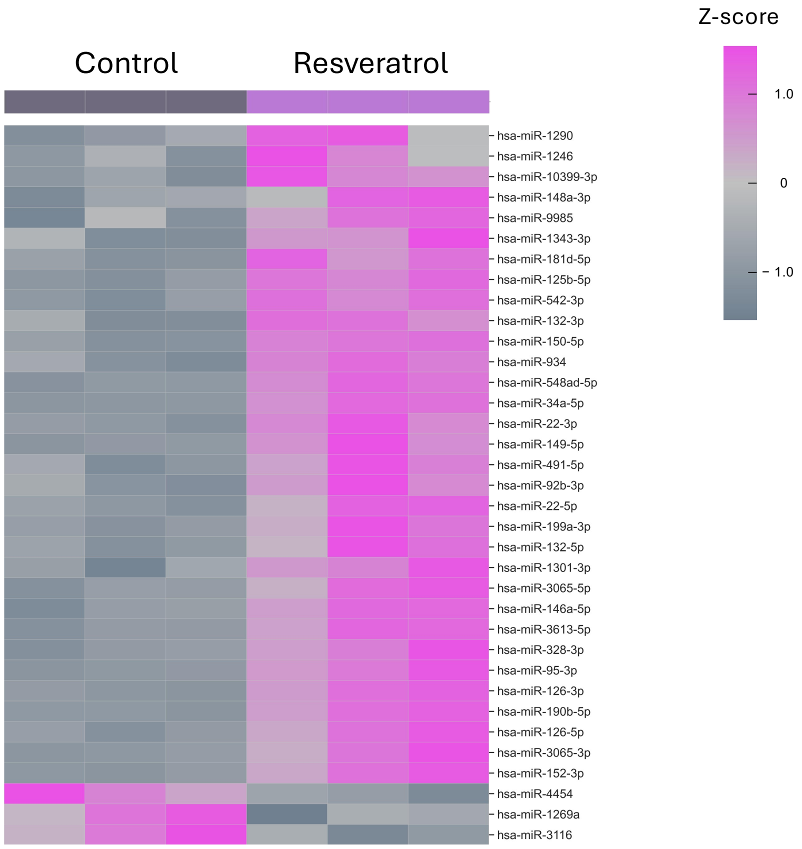

Figure S11. Heat map showing miRNAs whose expression levels differed by more than 1.5-fold in response to resveratrol treatment in HT29 cells (*P* < 0.05).

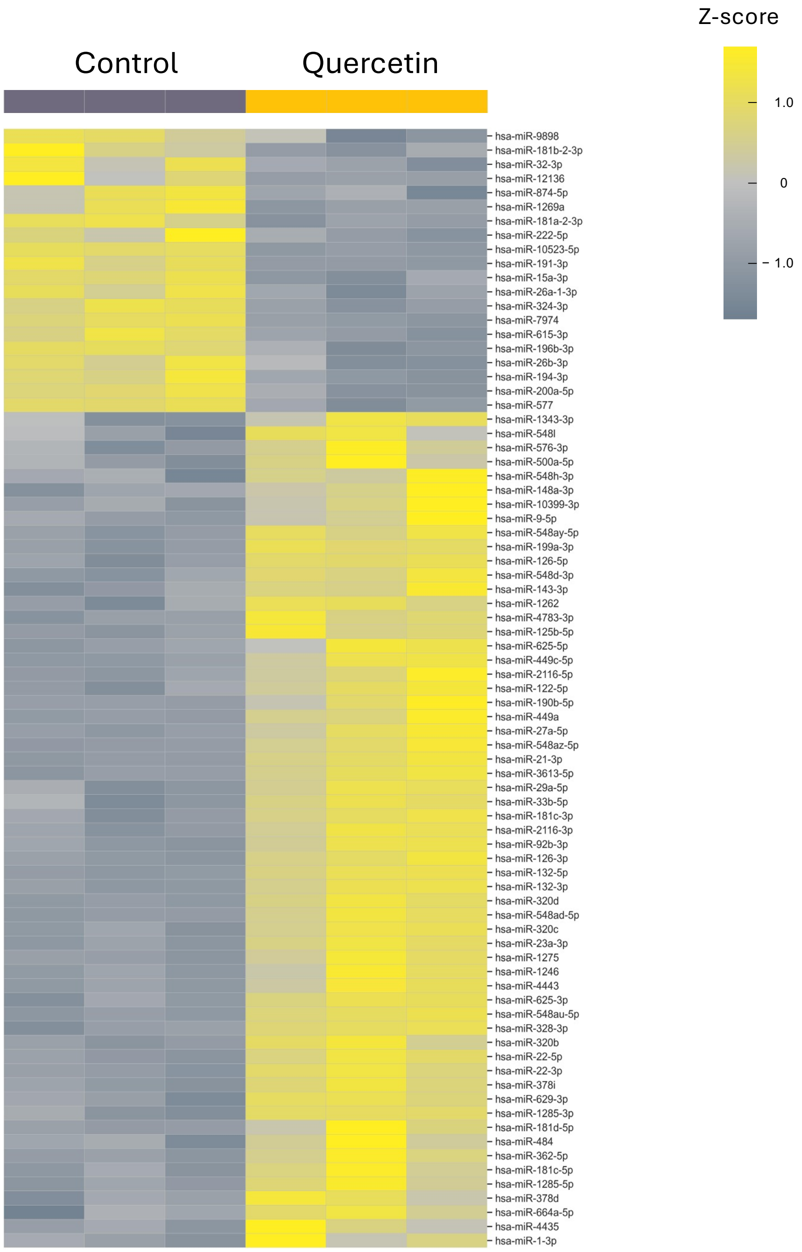

Figure S12. Heat map showing miRNAs whose expression levels differed by more than 1.5-fold in response to quercetin treatment in HT29 cells (*P* < 0.05).

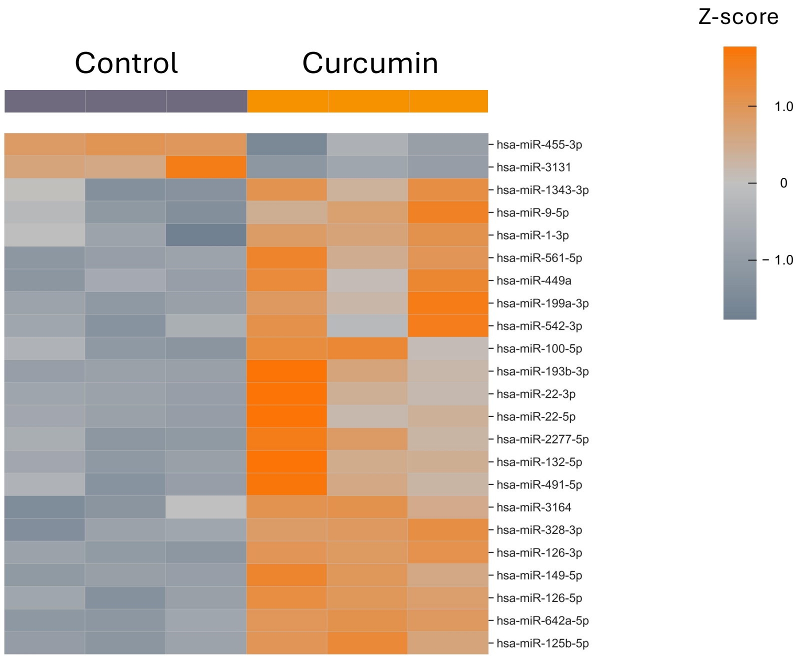

Figure S13. Heat map showing miRNAs whose expression levels differed by more than 1.5-fold in response to curcumin treatment in HT29 cells (*P* < 0.05).

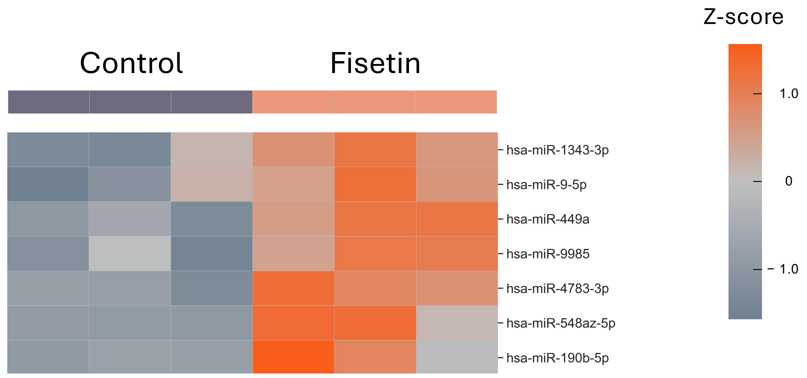

Figure S14. Heat map showing miRNAs whose expression levels differed by more than 1.5-fold in response to fisetin treatment in HT29 cells (*P* < 0.05).

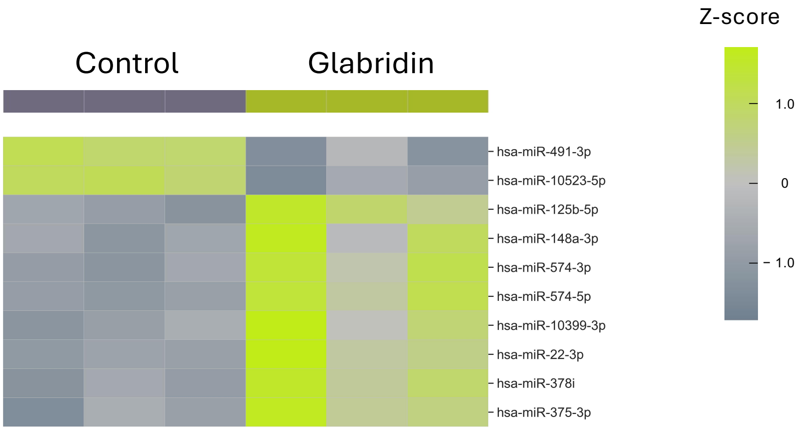

Figure S15. Heat map showing miRNAs whose expression levels differed by more than 1.5-fold in response to glabridin treatment in HT29 cells (*P* < 0.05).

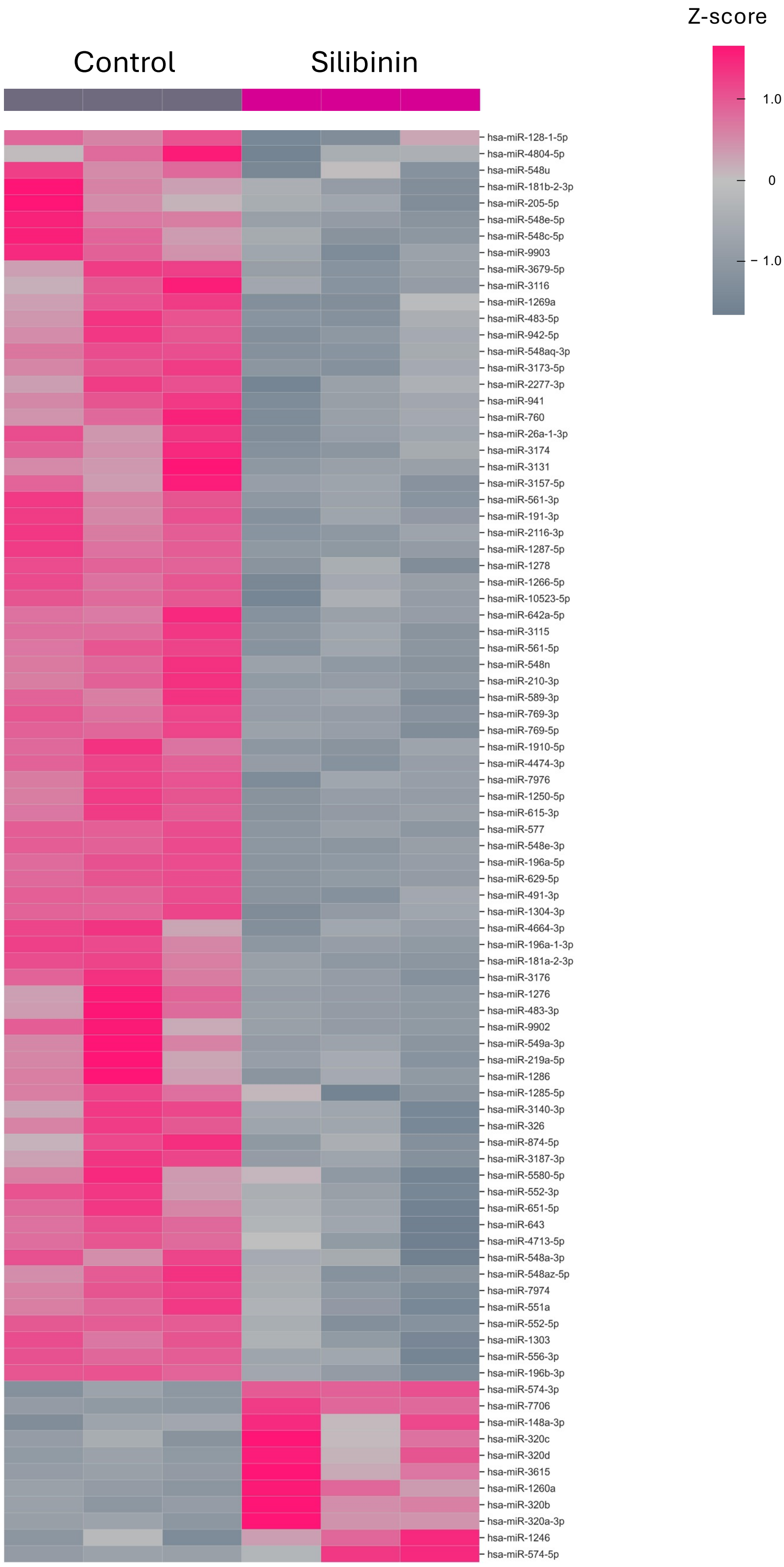

Figure S16. Heat map showing miRNAs whose expression levels differed by more than 1.5-fold in response to silibinin treatment in HT29 cells ($P < 0.05$).
